# The Chemists' and Druggists' Diary for 1875

**Published:** 1875-05

**Authors:** 


*The Chemists' and Druggists' Diary for* 1875, from the office of the London *Chemist and Druggist*, containing blank forms useful for the dispensing druggist.

